# Supplementary material for: VCP recruitment to mitochondria causes mitophagy impairment and neurodegeneration in models of Huntington's disease
Source: Nat Commun. 2016 Aug 26;7:12646. doi: 10.1038/ncomms12646 (PMC5007466; doi:10.1038/ncomms12646)
Supplement: Supplementary Information — Supplementary Figures 1-9, Supplementary Tables 1-2. [file ncomms12646-s1.pdf]

Reviewers' comments:

Reviewer #1 (Remarks to the Author):

This is a manuscript which investigated the role of VCP in mitochondrial dysfunction and its contribution to Huntington's disease pathogenesis. Mitochondria which accumulated VCP resulted in excessive mitophagy by recruiting the autophagosome component LC3 to the mitochondria which results in loss of mitochondrial mass and death of neurons. The authors developed a novel peptide termed HV-3 and showed that this abolishes VCP translocation to mitochondria and corrects excessive mitophagy and reduces cell death in both HD transgenic mouse and patient derived cells. It also was effective in HD mouse brains. It reduced behavioral and neuropathological phenotypes of HD in both fragments and full-length mutant huntingtin transgenic mice. They conclude that the peptide HV-3 may be a useful tool for development of new therapies for HD.

The initial studies were done on the HDHQ111 and HDHQ7 cell lines which have been widely utilized in Huntington's disease experimental cell culture models. Following immunoinfinity purification, they identified 9 proteins bound to mitochondria in the HDHQ111 cell lines and amongst these were VCP. They then validated this using by mass spectrometry.

Mitochondria isolated from transgenic mice and visualized in human postmortem brain tissue associated with VCP. Impressively HV-3 improved motor function and survival in transgenic mouse models of HD.

Overall these are well done and comprehensive studies. I however think that some of the figures are difficult to see and the demonstration of fragmented mitochondria and mitochondrial length reduction is not shown well. EM would be more convincing. Also how do the authors fit this in with the well established defects in PGC-1alpha in HD. Do you have to have both ?

Reviewer #2 (Remarks to the Author):

The manuscript by Guo et al demonstrated that VCP was translocated to mitochondria by selective association with mutant HTT, which in turn led to reduced mitochondria mass, shortened neurites and

cell death. Blocking the interaction of VCP and mutant HTT by a synthetic peptide, HV-3, abolished the translocation of VCP to mitochondria and reduced the phenotypes stated above. The authors showed chronic treatment with HV-3 significantly ameliorated disease pathology and behavioral deficits in both R6/2 and YAC128 mice.

These studies appeared to present significant advances in demonstrating that pathological interactions of mHTT and VCP contribute to excessive mitophagy in HD, and validating inhibition of such interactions could be beneficial in a variety of HD model systems including two mouse models. However, the key reagent used to perturb mHTT-VCP interactions is based on unproven rationales and poorly validated for specificity and PK/PD properties. Their cell based and in vivo studies are also of questionable quality and at least require additional studies to substantiate their claims.

1. The authors design two peptides to block protein-protein interactions, including HV-3 which is the critical reagent for majority of their cell-based and in vivo studies to block mHTT-VCP interactions on mitochondria. However, the principles used to design HV-3 are based on the assumption that primary amino acid sequence on these two proteins is likely the domain for interaction. This seems a very far-fetched idea, as protein-protein interaction residues, aside from a few well-characterized motifs, cannot be simply predicted based on primary amino acid sequence. The interacting amino acid residues of the two proteins often lies in the 3D structure and are often not contiguous on a given protein, that is why crystal structure or NMR is often needed to determine the precise interacting residues between the proteins. The principle used by the authors to design the inhibition peptide for mHTT-VCP interaction is simply unproven and unbelievable.

2. Much of the claims in the study are based on the HV-3 peptide, which is a fusion of cell-penetrating HIV TAT peptide and the HV-3 sequence based on the homology between HTT and VCP. The validity of the reagent as a specific and potent inhibitor of mHTT-VCP interaction is unclear. What is the binding affinity of HV-4 to mHTT or VCP? What is the IC50 of its inhibition of mHTT-VCP interaction? Given the small peptide sequence, what is its specificity? The latter may need proteomic study to identify all the brain proteins that may interact with the peptide, and to demonstrate that its affinity to VCP/HTT is much higher than other interacting proteins.

3. The in vivo use of TAT-tagged HV-3 peptide lacks essential PK/PD data. What is the PK/PD of the reagent with their osmotic pump delivery? The data in Supplemental Fig. 5B is simply unacceptable, with different background between control and HV-3, and with only a few cells shown and without any quantitation. Is there any long-term toxicity of such delivery? Did they measure adaptive immune

response to the HV-3 peptide? And if there is such a response, do the immune reactions rather than inhibition of mHTT-VCP interactions underlying therapeutic effects?

4. Given the questionable reagent to test their hypothesis, could the authors using additional, stronger evidence; to show functional perturbation is relevant to HD phenotypes in vivo? Can they demonstrate heterozygous mutation of VCP or subtle mutation of VCP selectively affect HTT interaction also modify HD mouse phenotypes? Can they overexpress or knockdown VCP in adult striatal or cortical neurons in HD mice to show phenotypic improvement?

5. Another major issue of the paper is very poor quality of much of the data. I am not sure how anyone can quantify subcellular organelles (mitochondria)/protein co-localization based on low power, low resolutions images --see Fig. 1D, 1F, 5E, Supplemental Fig. 2A?

6. The HD IPS cells were generated by the authors' group and published in a prior paper. These cells were not adequately characterized. What is their mHTT CAG length? Is there any karyotyping problem? The differentiation protocol used in Fig. 3E, with nearly 100% DARPP-32+ MSNs, is unheard-of. The authors need to explain why other labs, including HD iPSC Consortium, can only get 20-30% MSNs and they got such a high percentage.

7. The HD mouse studies are also raise serious concerns. The HV-3 peptide supposedly blocks VCP interaction with C-terminal of mHTT but it can reduce disease phenotypes in an mHTT exon1 model is not based on evidence. The authors' explanation is "toxic form of HTT (N-terminal fragment of HTT) undergoes conformation rearrangement leads to intramolecular proximity between the N domain and the polyproline region of HTT at the C-terminal (53, 54)" and then "it is possible that such a conformational change results in the binding of VCP to both the full-length and the fragment of HTT". This is utterly nonsensical, as the cited paper never showed mHTT exon1 has any conformational change to interact with the C-terminal domain of HTT where HV-3 is homologous to. The authors have to provide a new mechanism why this peptide is therapeutic in R6/2 mice.

8. The authors suggested that the mitochondrial pathology in mHTT expressing cells is resulted from enhanced mitophagosome production induced by the binding of VCP to LC3 on mitochondria. However, the authors fell short on just identifying the binding domain of VCP to LC3 in HeLa cells. It is

important to test whether mutation of this domain could abolish the mitopathic effect of overexpressing VCP in primary neurons.

9. In Fig. 4, the authors described that induced mitochondrial association of LC3 by expression of VCP in striatal cells could be inhibited by treatment with HV-3. However, their results do not support this conclusion. Similar ratio of mitochondria-associated GFP-LC3B in VCP-plasmids transfected over control-plasmids transfected cells are observed in HV-3 and control peptide treated cells. Overall LC3 levels in those cells are also needed to be verified. Furthermore, in Fig.4B, the experiments were performed in HeLa cells. Thus, the conclusion should not be generalized to striatal cells.

10. In Fig. 1F and S2A, the values of Pearson's efficiency for colocalization analyses should be presented without normalization. Based on the concept of Pearson's efficiency, it is not suitable to "normalize" the data to the control groups.

11. The proteomic data in Fig. 1a and Supplementary Fig. 1 is of questionable quality. Why are the VCP interactors in mitochondria fractions mainly cytoplasmic (Hsp90, Cct6a, Nestin, Serbp1) or nuclear proteins (Alyref)? A known mitochondrial VCP interactor, Parkin, is not found among their interactors. Given so few proteins were found (9), the use of the word "interactome" and list percentage of protein in function categories in Fig. 1a is misleading.

Response letter

RE: VCP recruitment to mitochondria by mutant Huntingtin causes mitophagy impairment and neurodegeneration in models of Huntington's disease (manuscript reference NCOMMS-16-00149)

In the following document, we first addressed the issues that the editor found particularly important, then provided our point-by-point responses to the specific comments of the individual reviewers.

RESPONSE TO THE COMMENTS FROM EDITOR:

"We expect to see extensive data on the molecular characterization of HV-3, as well as further convincing evidence to show that mHTT-VCP interaction is relevant for HD pathogenesis"

Response: Per the above comments, we have done the most extensive experiments within our power to provide further data on the molecular characterization of HV-3 and the relevance of the mtHtt and VCP interaction in human HD. The new data provided in the revised manuscript are summarized below. Due to the additional experiments and changes requested, we made a number of changes to the figures. These are described in detail in our point-by-point responses to the reviewers. The figures, related results, figure legends, and discussion as well as methods are all added in the appropriate places of the revised manuscript. All newly added sentences are underlined in the manuscript. We believe these changes have significantly improved our manuscript and we hope that the editor will now find the revised manuscript suitable for publication.

The molecular characterization of HV-3:

1) **We have provided new data showing the selectivity of HV-3 to block the interaction between VCP and mtHtt.** In the revised manuscript, we showed that the IC50 of HV-3 to inhibit the interaction of mtHtt and VCP in cultured cells is 2.11 µM (new Supplemental Figure 4C). Further, we provided data showing that treatment with HV-3 had no effects on the interaction between mtHtt and Tim23, a molecular event previously reported, and the interaction between VCP and its known binding protein UBXD1 (new Figure 3D). These data suggest the selectivity of HV-3 on inhibition of mtHtt and VCP binding. The results are presented on page 9 of the revised manuscript. Please also see our response to reviewer #2 comment 2.

2) **We have provided new data showing the binding affinity of HV-3 to VCP using VCP purified protein.** Our molecular docking analysis showed that HV-3 binds to the surface of the VCP structure (see supplemental Figure 4E in the original manuscript). For the revised manuscript, we performed isothermal titration calorimetry (ITC) with recombinantly expressed and purified full-length VCP to determine the binding affinity of HV-3 to VCP. Our analysis of the binding isotherms clearly showed that HV-3 binds to VCP with a K_d of 17.9 µM (new Figure 3E). The results are presented on page 9 of the revised manuscript. Please see our response to reviewer #2 comment 2.

3) **We have provided new data showing the specificity of HV-3 to VCP.** To further examine the specificity of HV-3 to VCP, we incubated biotin-conjugated HV-3 peptide and biotin-conjugated TAT (control peptide) with total protein lysates of HD mouse striatal cells and HD YAC128 mouse brains. We found that biotin-HV-3 but not biotin-TAT pulled down VCP in HD cells and HD animal brains. Moreover, biotin-HV-3 bound more strongly to VCP in HD mutant cells and HD YAC128 mouse brains than in wildtype counterparts. In contrast, we did not observe the binding between biotin-HV3 and a cytosolic protein Enolase nor between biotin-HV-3 and a mitochondrial protein Clpp (new Figure 3F). These new data further show that HV-3 binds to VCP specifically. The results are presented on pages 9 and 10 in the revised manuscript (see our response to reviewer #2 comment 2).

4) **We have provided new data showing the dependence of HV-3 efficacy on VCP**. In the cultured HdhQ111 cells (mutant cells), we knocked down VCP by VCP siRNA followed by treatment with peptide HV-3. We found that silencing VCP reduced mitochondrial depolarization in Hdh111Q cells relative to the cells with control siRNA. However, treatment with HV-3 had no additional protection on mitochondrial depolarization in the presence of VCP siRNA, even though HV-3 treatment promoted mitochondrial membrane potential in HdhQ111 cells treated with control siRNA (new Figure 4A). This finding indicates that the presence of VCP is required for HV-3 treatment

efficacy in HD models. The results are presented on page 10 in the revised manuscript (also see our response to reviewer #2 comment 2).

5) **We have provided new data showing that HV-3 is not toxic to naïve mice.** We performed a toxicity analysis of HV-3 in mice at Stanford University. The experimenter who conducted the analysis was blind to the treatment groups. Based on the results of the analysis, a four-week sustained treatment with HV-3 by pump implantation had no effects on blood chemistry, hematology, and gross necropsy of naïve mice when compared to mice treated with saline (new Suppl Figure 8). Moreover, a four-week treatment with HV-3 had minor effects on immuno-density of CD3, a marker of T-cell for adaptive immune response, in the brains and spleens of naïve mice. Treatment with HV-3 also had no effects on the size and weight of the spleens (new Suppl Figure 8). These results indicate that HV-3 treatment is nontoxic and may have minimal effects on adaptive immune response during the 4-week period of treatment. In our original manuscript, we showed that sustained 8-week treatment with HV-3 did not affect the survival, body weight or lifespan of wildtype mice and 9-month sustained treatment with HV-3 had minor effects on the behavioral status of wildytpe mice (Figures 6, 7 in the original manuscript, new Figures 7, 8 in the revised one). These results collectively suggest that HV-3 is well-tolerated in animals. The results are presented on page 15 in the revised manuscript. Please also see our response to the reviewer #2 comment 3.

Overall, these new data demonstrate the selectivity of HV-3 on inhibition of VCP/mtHtt binding, the specificity of HV-3 to VCP, and the lack of toxicity of HV-3 in animals.

The convincing evidence to show that mHTT-VCP interaction is relevant for HD pathogenesis:

1. **We have included new data showing the interaction between VCP and mtHtt on the mitochondria of HD patient fibroblasts.** We isolated mitochondria, ER, and cytosolic fractions from two HD patient fibroblasts and two normal subjects, and performed immunoprecipitation analysis to determine the interaction between VCP and mtHtt. We found that, consistent with our findings in HD mouse culture and HD mouse brains, VCP was bound to mtHtt on the mitochondria but not on the ER or cytosolic fractions in HD patient fibroblasts (new Figure 2C). The results are present on page 7 in the revised manuscript.

2. **We have included new data showing the interaction between VCP and mtHtt in postmortem brains of HD patients with different severities of the disease.** We obtained frozen postmortem cortical brain tissues from five HD patients and five normal subjects from Dr. Eliezer Masliah of the University of California at San Diego. Based on neuropathology reports of these five HD patients, two HD patients (5348 and 5263) exhibited severe brain atrophy and extensive neuronal loss, two HD patients (5298 and 5496) showed moderate brain atrophy and neuronal loss, and one HD patient (5374) had very subtle neuronal loss. We determined the VCP and mtHtt interactions in these samples by immunoprecipitation. We observed the binding between VCP and mtHtt in the cortical protein lysates of postmortem brain tissues of four HD patients (5348, 5263, 5298 and 5496) who exhibited moderate to severe neuronal loss and brain atrophy (new Figure 2D). In the HD patient with subtle neuronal loss (5374), we did not observe the interaction (new Suppl Figure 2D). The results are on page 7 in the revised manuscript.

Together, these findings in HD patient fibroblasts and HD patient postmortem brains not only support our observation that VCP/mtHtt binding is implicated in HD pathogenesis, but also suggest that the

interaction might be relevant to the severity of HD pathology. Please also see our response to reviewer #3 comments 4 and 5.

REVIEWER COMMENTS:

REVIEWER #1:

We thank the reviewer for the constructive comments and criticism and have made appropriate revisions according to the comments.

This is a manuscript which investigated the role of VCP in mitochondrial dysfunction and its contribution to Huntington's disease pathogenesis. Mitochondria which accumulated VCP resulted in excessive mitophagy by recruiting the autophagosome component LC3 to the mitochondria which results in loss of mitochondrial mass and death of neurons. The authors developed a novel peptide termed HV-3 and showed that this abolishes VCP translocation to mitochondria and corrects excessive mitophagy and reduces cell death in both HD transgenic mouse and patient derived cells. It also was effective in HD mouse brains. It reduced behavioral and neuropathological phenotypes of HD in both fragments and full-length mutant huntingtin transgenic mice. They conclude that the peptide HV-3 may be a useful tool for development of new therapies for HD.
The initial studies were done on the HDHQ111 and HDHQ7 cell lines which have been widely utilized in Huntington's disease experimental cell culture models. Following immunoinfinity purification, they identified 9 proteins bound to mitochondria in the HDHQ111 cell lines and amongst these were VCP. They then validated this using by mass spectrometry. Mitochondria isolated from transgenic mice and visualized in human postmortem brain tissue associated with VCP. Impressively HV-3 improved motor function and survival in transgenic mouse models of HD. Overall these are well done and comprehensive studies.

Comment 1: I however think that some of the figures are difficult to see and the demonstration of fragmented mitochondria and mitochondrial length reduction is not shown well. EM would be more convincing.

Response: Based on the reviewer's comment, we now provide new immunostaining images of mitochondrial morphology in the new Figure 4B of the revised manuscript. Following the reviewer's comment, we also performed an electron microscopy (EM) study to analyze mitochondrial morphology in HdhQ7 and HdhQ111 cells treated with either the control peptide TAT or the peptide inhibitor HV-3. As shown in the new Figure 4C in the revised manuscript, HdhQ111 cells (HD mutant cells) exhibited much shorter mitochondria when compared to that in wildtype HdhQ7 cells. The length of mitochondria in HdhQ111 cells were significantly increased by the treatment with HV-3. Again, treatment with HV-3 in wildtype HdhQ7 cells had minor effects on mitochondrial morphology. Moreover, we observed a great increase in the number of mitophagosome in the HdhQ111 cells treated with the control peptide. This new finding further supports our observation that HD is associated with excessive mitophagy. In contrast, treatment with HV-3 corrected the abnormalities of mitophagy. The new Figure 4C, figure legend and the results (pages 10 line 22 and page 11 line 1) are added in the revised manuscript. The method of EM analysis is now provided in the revised manuscript on page 25.

Comment 2: Also how do the authors fit this in with the well-established defects in PGC1-alpha in HD. Do you have to have both?

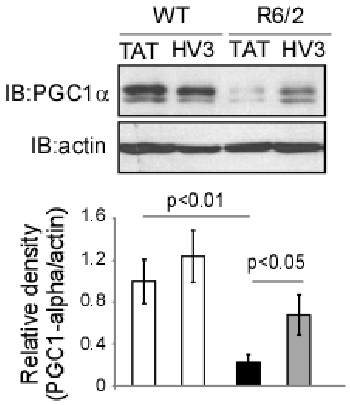

Response: We thank the reviewer for these insightful comments. Following the reviewer's suggestion, we determined the protein level of PGC1-alpha in the mouse brains of R6/2 mice treated with either HV-3 or control peptide TAT. Consistent with previous studies, we observed that PGC1-alpha was decreased in HD R6/2 mouse brains. To our surprise, treatment with HV-3 can partially improve the protein level of PGC1-alpha in the R6/2 mouse brains (see the figure on the right). It is possible that this increase in PGC1-alpha level is an indirect effect of improvement of mitochondrial mass and quality by the HV-3 treatment. Recent studies have shown that PGC1-alpha regulates mitophagy and autophagy via TFEB (Tsunemi T et al., Sci Trans Med, 2014; Siddiqui A et al., J Neurosci, 2015). Thus, our result may raise another possibility that there is a correlation between PGC1alpha and VCP-mediated mitophagy in HD. However, the mechanism remains to be determined, and it is beyond the scope of this manuscript. In response to the reviewer's comment, we now add the following sentences in the discussion in the revised manuscript.

"In addition, PGC1α, a key regulator of mitochondrial biogenesis implicated in HD pathogenesis, has recently been reported to regulate mitophagy and autophagy through TFEB signaling [65, 66]. It would be interesting to determine whether there is a cross talk between PGC1α and VCP-mediated mitophagy in HD." (page 18 line 19)

REVIEWER #2:

We thank the reviewer for constructive criticism and have made appropriate revisions according to the comments.

Comment 1: The authors design two peptides to block protein-protein interactions, including HV-3 which is the critical reagent for majority of their cell-based and in vivo studies to block mHTT-VCP interactions on mitochondria. However, the principles used to design HV-3 are based on the assumption that primary amino acid sequence on these two proteins is likely the domain for interaction. This seems a very far-fetched idea, as protein-protein interaction residues, aside from a few well-characterized motifs, cannot be simply predicted based on primary amino acid sequence. The interacting amino acid residues of the two proteins often lies in the 3D structure and are often not contiguous on a given protein, that is why crystal structure or NMR is often needed to determine the precise interacting residues between the proteins. The principle used by the authors to design the inhibition peptide for mHTT-VCP interaction is simply unproven and unbelievable.

Response: Two non-related proteins that interact in an inducible manner often have shared short sequences of homology that could represent binding sites of both inter- and intra-molecular interactions (1-4). A peptide corresponding to that sequence can serve as decoys for one of the proteins, preventing the binding of that protein to the target protein (1-3, 5). Although peptides represent only a part of the

interaction surface, previous studies including ours showed that short peptides derived from the interaction sites between two proteins act as highly specific inhibitors and are effective agents in basic research and in animal models of human diseases, such as neurological disorders, cardiovascular diseases, cancer, and diabetes (5-14). Our lab has used the rationally designed short peptide inhibitor P110 which is a peptide inhibitor that interferes with the mitochondrial fission protein Drp1 and its binding protein, Fis1 (14). We used P110 as a pharmacological tool to identify the role of these proteins in mitochondrial signal transduction in HD (15) and other disease models (6-8). A Phase I clinical trial on the efficacy of peptide P110 in patients with neurodegenerative diseases is scheduled to begin later in 2016. Other peptides were generated to study the functional consequences of protein kinase C [PKC (16, 17)] by the Mochly-Rosen lab where I completed my post-doctoral training at Stanford University. These peptides have been used by many laboratories for *in vitro* and *in vivo* testing of a variety of species including humans and have resulted in over 250 publications. Thus, we have had considerable success in designing, synthesizing, and characterizing peptide inhibitors that are highly specific for the targeted protein-protein interaction.

In addition to the efficacy of HV-3 in both HD cell cultures and HD transgenic mouse models shown in the original manuscript, in the revised manuscript, we provide new data further demonstrating the selectivity of HV-3 in blocking mtHtt/VCP binding, the affinity and specificity of HV-3 on VCP, and the lack of toxicity of HV-3 in animals. The references cited above are listed at the end of this section. We hope that these lines of evidence could convince the reviewer that the HV-3 is at least a useful pharmacologic tool to examine the functional roles of mitochondria-accumulated VCP and mtHtt/VCP binding in HD models.

1. Ron D, and Mochly-Rosen D. An autoregulatory region in protein kinase C: the pseudoanchoring site. *Proceedings of the National Academy of Sciences of the United States of America.* 1995;92(2):492-6.
2. Qvit N, and Mochly-Rosen D. Highly Specific Modulators of Protein Kinase C Localization: Applications to Heart Failure. *Drug discovery today Disease mechanisms.* 2010;7(2):e87-e93.
3. Souroujon MC, and Mochly-Rosen D. Peptide modulators of protein-protein interactions in intracellular signaling. *Nature biotechnology.* 1998;16(10):919-24.
4. Kheifets V, and Mochly-Rosen D. Insight into intra- and inter-molecular interactions of PKC: design of specific modulators of kinase function. *Pharmacol Res.* 2007;55(6):467-76.
5. Churchill EN, Qvit N, and Mochly-Rosen D. Rationally designed peptide regulators of protein kinase C. *Trends Endocrinol Metab.* 2009;20(1):25-33.
6. Guo X, Sesaki H, and Qi X. Drp1 stabilizes p53 on the mitochondria to trigger necrosis under oxidative stress conditions in vitro and in vivo. *Biochem J.* 2014;461(1):137-46.
7. Su YC, and Qi X. Inhibition of excessive mitochondrial fission reduced aberrant autophagy and neuronal damage caused by LRRK2 G2019S mutation. *Hum Mol Genet.* 2013;22(22):4545-61.
8. Disatnik MH, Ferreira JC, Campos JC, Gomes KS, Dourado PM, Qi X, and Mochly-Rosen D. Acute inhibition of excessive mitochondrial fission after myocardial infarction prevents long-term cardiac dysfunction. *J Am Heart Assoc.* 2013;2(5):e000461.
9. Inagaki K, Chen L, Ikeno F, Lee FH, Imahashi K, Bouley DM, Rezaee M, Yock PG, Murphy E, and Mochly-Rosen D. Inhibition of delta-protein kinase C protects against reperfusion injury of the ischemic heart in vivo. *Circulation.* 2003;108(19):2304-7.
10. Qi X, Inagaki K, Sobel RA, and Mochly-Rosen D. Sustained pharmacological inhibition of deltaPKC protects against hypertensive encephalopathy through prevention of blood-brain barrier breakdown in rats. *The Journal of clinical investigation.* 2008;118(1):173-82.
11. Palaniyandi SS, Sun L, Ferreira JC, and Mochly-Rosen D. Protein kinase C in heart failure: a therapeutic target? *Cardiovascular research.* 2009;82(2):229-39.
12. Kim J, Choi YL, Vallentin A, Hunrichs BS, Hellerstein MK, Peehl DM, and Mochly-Rosen D. Centrosomal PKCbetaII and pericentrin are critical for human prostate cancer growth and angiogenesis. *Cancer Res.* 2008;68(16):6831-9.
13. Budas GR, Churchill EN, and Mochly-Rosen D. Cardioprotective mechanisms of PKC isozyme-selective activators and inhibitors in the treatment of ischemia-reperfusion injury. *Pharmacol Res.* 2007;55(6):523-36.
14. Qi X, Qvit N, Su YC, and Mochly-Rosen D. A novel Drp1 inhibitor diminishes aberrant mitochondrial fission and neurotoxicity. *J Cell Sci.* 2013;126(Pt 3):789-802.
15. Guo X, Disatnik MH, Monbureau M, Shamloo M, Mochly-Rosen D, and Qi X. Inhibition of mitochondrial fragmentation diminishes Huntington's disease-associated neurodegeneration. *J Clin Invest.* 2013;123(12):5371-88.
16. Souroujon MC, and Mochly-Rosen D. Peptide modulators of protein-protein interactions in intracellular signaling. *Nat Biotechnol.* 1998;16(10):919-24.

17. Chen L, Wright LR, Chen CH, Oliver SF, Wender PA, and Mochly-Rosen D. Molecular transporters for peptides: delivery of a cardioprotective epsilonPKC agonist peptide into cells and intact ischemic heart using a transport system, R(7). *Chem Biol.* 2001;8(12):1123-9.

Comment 2: Much of the claims in the study are based on the HV-3 peptide, which is a fusion of cell-penetrating HIV TAT peptide and the HV-3 sequence based on the homology between HTT and VCP. The validity of the reagent as a specific and potent inhibitor of mHTT-VCP interaction is unclear. What is the binding affinity of HV-3 to mHTT or VCP? What is the IC50 of its inhibition of mHTT-VCP interaction? Given the small peptide sequence, what is its specificity? The latter may need proteomic study to identify all the brain proteins that may interact with the peptide, and to demonstrate that its affinity to VCP/HTT is much higher than other interacting proteins.

Response: Based on the reviewer's comments, we provide the following data in the revised manuscript to show the selectivity of HV-3 on inhibition of mtHtt/VCP binding, the affinity and specificity of HV-3 on VCP, and the IC50 of HV-3 to block mtHtt/VCP interaction in culture.

1) The IC50 of HV-3 to inhibit the interaction of mtHtt and VCP in the cultured cells is 2.11 µM (see new Supplemental Figure 4C and the results on page 9 line 4 in the revised manuscript). Further, we found that treatment with HV-3 had no effects on the interaction between mtHtt and Tim23, a molecular event previously reported, or on the interaction between VCP and its known binding partner UBXD1 (see new Figure 3D). These data suggest a selectivity of HV-3 for inhibition of mtHtt and VCP binding (see results on page 9 line 8 in the revised manuscript).

2) Our molecular docking analysis showed that HV-3 binds to the surface of the VCP structure (see supplemental Figure 4E in the original manuscript). In the revised manuscript, we performed isothermal titration calorimetry (ITC) with recombinantly expressed and purified full-length VCP to determine the binding affinity of HV-3 to VCP. As shown in the new Figure 3E of the revised manuscript, our analysis of the binding isotherms clearly shows that HV-3 binds to VCP with a K_d of 17.9 µM. The results for the above data are on page 9 line 15 of the revised manuscript.

3) In the cultured HdhQ111 cells, we knocked down VCP by VCP siRNA followed by treatment with peptide HV-3. We found that silencing VCP reduced mitochondrial depolarization in Hdh111Q cells relative to the cells with control siRNA. However, treatment with HV-3 had no additional protective effect on mitochondrial depolarization in the presence of VCP siRNA, even though HV-3 treatment promoted mitochondrial membrane potential in HdhQ111 cells treated with control siRNA (new Figure 4A-right panel in the revised manuscript). This finding demonstrates that the presence of VCP is required for HV-3 efficacy in HD models. The results are now on page 10 line 20 of the revised manuscript.

4) To further examine the specificity of HV-3 to VCP, we incubated biotin-conjugated HV-3 peptide and biotin-conjugated TAT (control peptide) with total protein lysates of HD mouse striatal cells and HD YAC128 mouse brains. We found that biotin-HV-3 but not biotin-TAT pulled down VCP in HD cells and HD animal brains. Moreover, biotin-HV-3 was bound more strongly to VCP in HD mutant cells and HD YAC128 mouse brains than in wildtype counterparts (new Figure 3F in the revised manuscript). In contrast, no detectable binding was observed between biotin-HV3 and a cytosolic protein enolase or between biotin-HV-3 and a mitochondrial protein Clpp (new Fig. 3F in the revised manuscript), suggesting a specificity of HV-3. These results are found on page 9 line 20 of the revised manuscript.

The new figures above and related figure legends have all been added to the revised manuscript. The related discussion has been added on page 17 line 2 and line 5. The methods of ITC and immunoprecipitation with biotin-peptides have been added to the Method section (see pages 21-23) of the revised manuscript.

Comment 3: The in vivo use of TAT-tagged HV-3 peptide lacks essential PK/PD data. What is the PK/PD of the reagent with their osmotic pump delivery? The data in Supplemental Fig. 5B is simply unacceptable, with different background between control and HV-3, and with only a few cells shown and without any quantitation. Is there any long-term toxicity of such delivery? Did they measure adaptive immune response to the HV-3 peptide? And if there is such a response, do the immune reactions rather than inhibition of mHTT-VCP interactions underlying therapeutic effects?

Response: For the analysis of PK/PD, we have to subcontract with bio-company. The cost will be a minimum of $80,000. Due to limited funds, we cannot afford such an expensive experiment. Working with the Mass Spectrometry Core Facility at Cleveland Clinic Foundation Research Center, we attempted to determine the concentration of HV-3 after treatment in mice by pump implantation. We found that the peptide HV-3 can be detected in samples of total brain lysates, plasma, and the precipitated proteins from plasma. The peptide was not detectable in the supernatant of plasma after all of the proteins were removed by protein precipitation methods (see figure below). These data strongly suggest that the peptide binds to proteins in mice after treatment. The data seem to be consistent with our findings that HV-3 binds to VCP. Because of current technical constraints to dissociate peptide from the binding proteins, we were not able to test the accurate concentration of the peptide HV-3 in brain tissues and plasma. Therefore, we decided not to add these data to our manuscript. We would appreciate it if the reviewer could understand.

Based on the reviewer's comment, in the revised manuscript, we removed the original supplemental Fig. 5B.

Regarding toxicity of HV3, we performed toxicity analysis of HV-3 in mice at Stanford University. The experimenter who conducted the analysis was blind to the treatment groups. We found that a four-week treatment with HV-3 by pump implantation had no significant effects on blood chemistry, hematology, and gross necropsy of naïve mice when compared to mice treated with saline (new Suppl Figure 8 in the revised manuscript). Moreover, a four-week treatment with HV-3 had minor effects on immuno-density of CD3 (a marker of T-cells for adaptive immune response) in the brains and spleens of naïve mice. Treatment with HV-3 had no effects on the size and weight of the spleens (new Suppl Figure 8 in the revised manuscript). These results indicate that HV-3 treatment might have minor effects on adaptive immune response, at least during the period of 4-week sustained treatment. Together with the findings that 8-week treatment with HV-3 did not affect the survival, body weight and lifespan of wildtype mice and 9-month sustained treatment with HV-3 had minor effects on behavioral status of wildytpe mice in our original manuscript (new Figures 7, 8 in the revised manuscript), these results collectively demonstrate that HV-3 is nontoxic to animals.

The figures (new Suppl Figure 8) and figure legend have been added in the Supplemental information of the revised manuscript. The results of the above data are presented on page 15 line 5 of the revised manuscript.

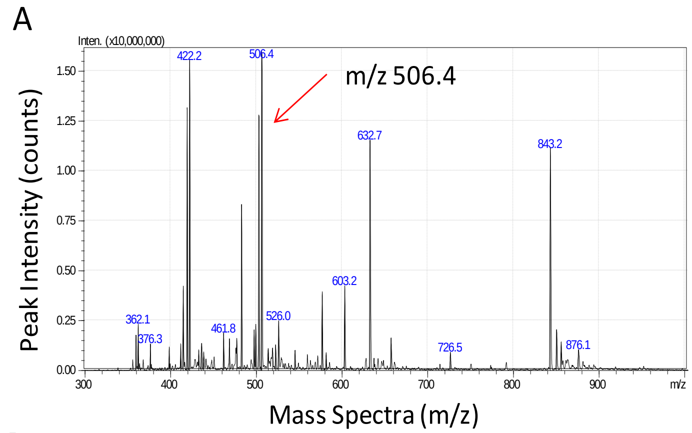

Mass Spectrometry analysis of HV-3 in mice. **(A)** Mass Spectra of Standard HV-3. HV-3 showed multiple charged ions in positive electrospray ionization and the ion at 5 charged (m/z 506.4) has the highest intensity on the Shimadzu LC/MS 8050. **(B)** HV-3 at 5 charged (m/z 506.4) in positive electrospray ionization was monitored. Shown is the LC/MS chromatograms of HV-3 (1000 nM) in mouse brain total lysates that were not subjected to protein precipitation. The possible HV-3 could be observed one day, one week and two weeks after treatment. **(C)** Shown is the LC/MS chromatograms of HV-3 (1000 nM) in mouse plasma. Upper: the plasma without protein precipitation; Middle: Supernatant after protein precipitation; Lower: Samples of precipitated proteins of plasma. The possible HV-3 can only be detected in total plasma and the sample

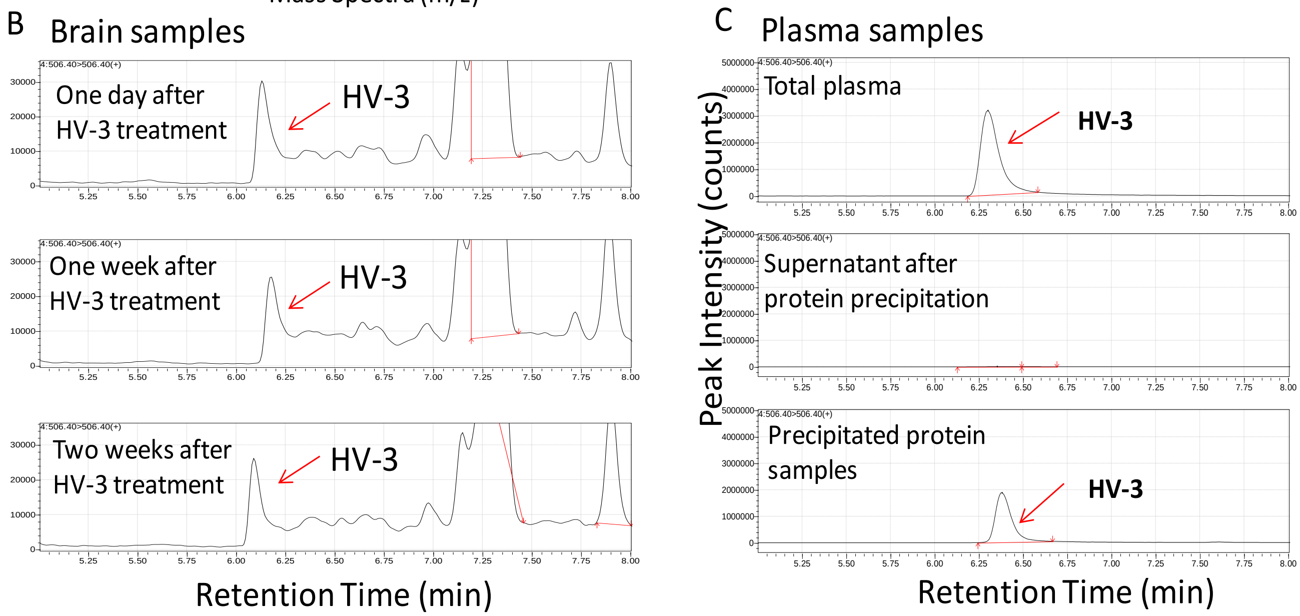

Comment 4: Given the questionable reagent to test their hypothesis, could the authors using additional, stronger evidence; to show functional perturbation is relevant to HD phenotypes in vivo? Can they demonstrate heterozygous mutation of VCP or subtle mutation of VCP selectively affect HTT interaction also modify HD mouse phenotypes? Can they overexpress or knockdown VCP in adult striatal or cortical neurons in HD mice to show phenotypic improvement?

Response: We thank the reviewer for the suggestion. HD is chronic and progressive. The HD transgenic mice (such as YAC128 mice or BACHD mice) will take a year to exhibit neuropathology. Moreover, the key amino acid of VCP that influences its interaction with Htt is not known. Thus, identification of heterozygous or subtle mutations of VCP selectively affecting Htt interaction and generation of a new HD mouse line as suggested by the reviewer is a lengthy process which is impossible to complete within the limited time of revision. The overexpression or knockdown of VCP in HD mice

may raise the possibility that such genetic regulation of VCP in HD mouse brains would influence VCP at basal levels or the activation of VCP on other subcellular organelles, such as ER, which may generate confusion. In the revised manuscript, we have included new data that demonstrates the specificity and affinity of HV-3 to VCP, the selectivity of HV-3 to inhibit the interaction between mtHtt and VCP, and the lack of toxicity of HV-3 in animals (See our responses to comments 2 and 3). Moreover, in the original manuscript, we showed that HV-3 treatment mainly influenced mitochondria-associated VCP while it had no effects on ER stress. The peptide HV-3 also had no effects on VCP mitochondrial level, mitochondrial morphology, or cell survival under basal conditions in culture and in animals. Overall, our findings suggest that HV-3 is a useful tool for us to investigate the functional role of VCP on mitochondria in HD models in culture and in animals.

Comment 5: Another major issue of the paper is very poor quality of much of the data. I am not sure how anyone can quantify subcellular organelles (mitochondria)/protein co-localization based on low power, low resolutions images --see Fig. 1D, 1F, 5E, Supplemental Fig. 2A?

Response: In the revised manuscript, we replaced the images with higher resolution images. In new Figure 1D, 1G (old 1F) and Suppl Fig. 2A, we also include inserts that show the enlarged images. In new Figure 6E (old Figure 5E), we used the co-localization between Flag-VCP and Tom20 to only indicate the cells expressing Flag-VCP.

Comment 6: The HD IPS cells were generated by the authors' group and published in a prior paper. These cells were not adequately characterized. What is their mHTT CAG length? Is there any karyotyping problem? The differentiation protocol used in Fig. 3E, with nearly 100% DARPP-32+ MSNs, is unheard-of. The authors need to explain why other labs, including HD iPSC Consortium, can only get 20-30% MSNs and they got such a high percentage.

Response: The CAG repeats of the HD patient line are 41 and 19 based on our unpublished data. We previously showed (Guo et al., J Clin Invest 2013) that the yield of DARPP-32 positive neurons was approximately 15%, which is consistent with the previous study *(Zhang N, An MC, Montoro D, Ellerby LM., Plos Curr., 2010)*. To better quantitate the neurite lengths of individual neurons, we reduced the culture density of neurons 20 days after differentiation by re-plating cells.

The number of DARPP32 neurons shown in the images of the original manuscript does not reflect the differentiation percentage of neurons. To collect the images of DARPP32+ neurons for quantitation, we only picked the area with a high number of DARPP32+ neurons. In our study, we only quantitated the neurons immuno-positive for both DARPP32, a marker of MSN, and Tuj1, a marker of neuron. The quantitation was performed by an observer blind to experimental conditions. The image shown on the right illustrates the differentiation percentage of the DARPP32/Tuj1 positive neurons derived from HD patient-iPS cells using our protocol.

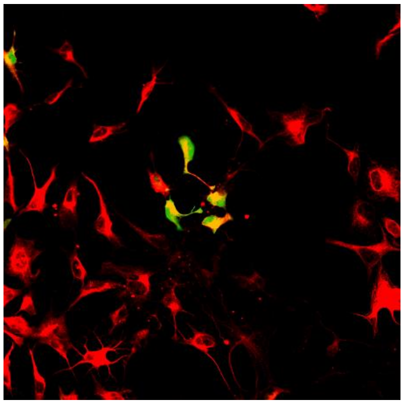

Neuronal cells derived from HD patient iPS cells. Neuronal differentiation was conducted in HD patient iPS cells using established protocol. On 20 days after the initiation of neuronal differentiation, cells (about 5,000) were re-plated on coverslips. In the image shown above, we stained cells with anti-DARPP32 (green, a marker of MSN) and antiTuj1 (red, a marker of neuron) antibodies to label neurons. This staining was performed 3 days after replating. The yield of DARPP32+/Tuj1+ neurons is approximately 15% overall using our protocol.

In response to the reviewer’s comments, we added the following sentences (underlined) in the **Method section** to further specify our methods for culturing and quantitation in the revised manuscript.

"iPS cells from a normal subject and a HD patient (carrying 41 CAG repeats) were differentiated into neurons using the protocol from our previous studies [5]. Twenty days after the initiation of differentiation, neurons (about 5,000 cells) were plated onto 12-mm poly-D-lysine/laminine-coated coverslips and grown in 24-well plates in neuronal differentiation medium as described previously [5]." (Page 21 and line 1)

"The quantitation of neurite length was conducted only in the neurons immuno-positive for both DARPP-32 and Tuj1 (neuron-specific class III beta-tubulin, 1:2000, Covance)." (Page 24 line 19).

Comment 7: The HD mouse studies are also raise serious concerns. The HV-3 peptide supposedly blocks VCP interaction with C-terminal of mHTT but it can reduce disease phenotypes in an mHTT exon1 model is not based on evidence. The authors' explanation is "toxic form of HTT (N-terminal fragment of HTT) undergoes conformation rearrangement leads to intramolecular proximity between the N domain and the polyproline region of HTT at the C-terminal (53, 54)" and then "it is possible that such a conformational change results in the binding of VCP to both the full-length and the fragment of HTT". This is utterly nonsensical, as the cited paper never showed mHTT exon1 has any conformational change to interact with the C-terminal domain of HTT where HV-3 is homologous to. The authors have to provide a new mechanism why this peptide is therapeutic in R6/2 mice.

Response: In response to the reviewer's comment, in the revised manuscript, we added a new discussion. The following sentences were added and underlined in the revised manuscript (page 17 line 11). The new references are provided in the revised manuscript.

"We found that HV-3 can block VCP accumulation on mitochondria and provide neuroprotection in HD R6/2 mice in which an N-terminal mtHtt fragment is expressed. VCP has been shown to bind to Htt exon 1 fragment with expanded polyQ via the polyQ tract sequence and co-localize with mtHtt in perinuclear cytoplasmic region of neurons in R6/2 mice [26]. Because N-terminal mtHtt fragments can co-localize and co-aggregate with normal Htt fragments [59], it is possible that VCP, mtHtt fragment and endogenous Htt form a protein complex in HD R6/2 mice. HV-3 may block VCP accumulation on the mitochondria in R6/2 mice by disrupting the interaction of the complex, thus reducing subsequent mitochondrial damage. However, other mechanisms may exist, which remain to be further investigated."

Comment 8: The authors suggested that the mitochondrial pathology in mHTT expressing cells is resulted from enhanced mitophagosome production induced by the binding of VCP to LC3 on mitochondria. However, the authors fell short on just identifying the binding domain of VCP to LC3 in HeLa cells. It is important to test whether mutation of this domain could abolish the mitopathic effect of overexpressing VCP in primary neurons.

Response: Based on the reviewer's comment, we expressed the wildtype VCP and two VCP LIR mutants in the primary striatal neurons. We determined the mitochondrial morphology and neurite length of medium spiny neurons. We found that similar to the findings in Hela cells, neurons expressing VCP

LIR mutants VCP-FV^{AA} and VCP-YI^{AA} exhibited fewer mitochondrial aggregates when compared to those in neurons expressing VCP WT. Moreover, neurons with these VCP LIR mutants showed longer neurites of medium spiny neurons than those in neurons with VCP WT. The results are presented on the page 14 line 5 of the revised manuscript. The new Figure 6E-H and related figure legends have also been included in the revised manuscript.

Comment 9: In Fig. 4, the authors described that induced mitochondrial association of LC3 by expression of VCP in striatal cells could be inhibited by treatment with HV-3. However, their results do not support this conclusion. Similar ratio of mitochondria-associated GFP-LC3B in VCP-plasmids transfected over control-plasmids transfected cells are observed in HV-3 and control peptide treated cells. Overall LC3 levels in those cells are also needed to be verified. Furthermore, in Fig.4B, the experiments were performed in HeLa cells. The conclusion should not be generalized to striatal cells.

Response: In the revised manuscript, we have included data that demonstrate that HV-3 treatment did not affect the GFP-LC3B total protein levels in the presence or absence of the VCP plasmid (See new Suppl Fig. 6C). The result is present on page 12 line 7 of the revised manuscript.

In original Fig. 4B, the experiments were performed in HdhQ7 striatal cells. It showed in the result section of original manuscript. We apologize for this mistake. Now, it is corrected in the figure legend of new Fig. 5B in the revised manuscript.

Comment 10: In Fig. 1F and S2A, the values of Pearson's efficiency for colocalization analyses should be presented without normalization. Based on the concept of Pearson's efficiency, it is not suitable to "normalize" the data to the control groups.

Response: Following the reviewer's comment, we have now provided new quantitation for the confocal imaging with raw values of Pearson's efficiency.

Comment 11: The proteomic data in Fig. 1a and Supplementary Fig. 1 is of questionable quality. Why are the VCP interactors in mitochondria fractions mainly cytoplasmic (Hsp90, Cct6a, Nestin, Serbp1) or nuclear proteins (Alyref)? A known mitochondrial VCP interactor, Parkin, is not found among their

interactors. Given so few proteins were found (9), the use of the word "interactome" and list percentage of protein in function categories in Fig. 1a is misleading.

Response: The proteomic analysis examined the interactors of Htt on the mitochondria in HD striatal cells. As shown in the figure legend of supplementary Figure 1C in the original manuscript, we listed the proteins in the table that only bind to mtHtt in HdhQ111 striatal cells. Among these protein candidates, VCP has been demonstrated to associate with mitochondria in our current study, Hsp90 has previously been shown to translocate to the mitochondria (Budas G et al., Cardiovascular Res, 2010). Nestin has recently been reported to be localized on the mitochondria (Wang J et al., Oncogene, 2015). At present, we cannot exclude the possibility that other protein candidates may translocate to or associate with the mitochondria.

In the study, we used HdhQ7 and HdhQ111 striatal cells to profile the interactors of mtHtt on mitochondria. As shown in our supplemental Figure 7G in the original manuscript, Parkin is expressed in neither HdhQ7 nor HdhQ111 cells. Thus, Parkin is not found in the interactors of mtHtt on the mitochondria in our proteomic analysis.

Based on the reviewer's comment, we changed the word "interactome" to "interactors of mtHtt". The change is underlined in the revised manuscript (pages 5, 35).

Reviewer #3:

We thank the reviewer for constructive criticism and have made appropriate revisions according to the comments.

Guo and colleagues studied the molecular links between valosin containing protein (VCP) and mitochondria in Huntington's disease (HD) neurons. Authors extensively studied VCP interaction with mutant huntingtin (mHtt), particularly on mitochondria. Authors found VCP translocation to mitochondria in mutant Htt neurons, and facilitate mitochondrial loss through increased mitophagy by recruiting autophagosome component LC3 to mitochondria. They also claimed HV-3 peptide inhibited VCP translocation to mitochondria by reducing mHtt interactions. These observations are interesting. Authors used several reagents, mouse models and progenitor striatal WT (Q7/Q7) and mutant

(Q111/Q111) neurons from knockin mice. Authors are well qualified to perform all experiments conducted in this study. Used methods are acceptable. Although VCP is reported to interact with mutant neurodegenerative diseases proteins, including mHtt by several groups (Hirabayashi et al 2001, Almeida et al 2015 and Mori et al 2013) - the present study findings are still novel and interesting for HD and other neurodegenerative disease(s) researchers. If present study findings are reproducible, VCP molecular links with mutant proteins, and their associations with mitophagy are highly significant. Overall, it is a well-done and well executed and well-presented manuscript.

Comment 1: It is important to demonstrate VCP translocation to mitochondria using immunogold electron microscopy

Response: Per Reviewer's suggestion, we performed immunogold electron microscopy to examine the translocation of VCP to the mitochondria in HD striatal cells. We found that in HdhQ111 cells, there were more VCP-immunopositive gold particles accumulated on the surface of the mitochondria than that in wildtype HdhQ7 cells, further demonstrating VCP translocation to mitochondria in HD. This finding is consistent with our observations using confocal imaging and western blot analysis of subcellular fractionations that VCP is translocated to the mitochondria in HD models in culture, animals and patient brains. The figure (new Figure 1E) and the following sentences are added in the revised manuscript.

"Immunogold electron microscopy (EM) found more particles of immuno-labeled VCP localized on the surface of mitochondria in HdhQ111 cells than that in HdhQ7 cells (Fig. 1E)." (page 6 line 6)

The figure legend of the new Figure 1E is added in the revised manuscript, the method of immunogold electron microscopy is now provided in the Method section.

Comment 2: HV-3 peptide story is not convincing at all (it may be worth reporting separately)

Response: In the revised manuscript, we performed extensive studies to characterize the molecular action of HV-3. *First*, we found that HV-3 blocked the interaction between mtHtt and VCP, and had no effects on other protein-protein interactions (new Figure 3D), indicating its selectivity. *Second*, we

showed that HV-3 bound to VCP purified recombinant protein with a binding affinity at a Kd of 17.9 µM, and that biotin-HV-3 pulled down VCP but did not bind to either a cytosolic protein Enolase or a mitochondrial protein Clpp (new Figures 3E and F). These findings suggest a specificity of HV-3. *Third*, we observed that HV-3 had no additional protection on mitochondrial depolarization in HD cells in the presence of VCP siRNA (new Figure 4A), indicating that the presence of VCP is required for HV-3 treatment efficacy. *Finally*, we provided data of toxicity analysis of HV-3 and showed that HV-3 treatment had no significant effects on blood chemistry, hematology, and gross necropsy of naïve mice. Moreover, HV-3 had no effects on the immunodensity of CD3, a marker of T-cell-related adaptive immune response (new Suppl Figure 8 in the revised manuscript). These results demonstrate that HV-3 is nontoxic in animals. Together with our results presented in the original manuscript, we hope that these new data on the molecular characterization of HV-3 could convince the reviewer that the peptide HV-3 is a useful pharmacologic tool to determine the selective role of VCP on mitochondria in HD models in culture and in animals. Please also refer our responses to the editor and reviewer #2 comments 2 and 3.

Comment 3: In the introduction, authors mentioned we recently profiled mitochondrial interactome of mHtt and identified VCP as a high abundance mHtt interacting protein - it is important to show the publication/data;

Response: The list of proteins that bind to mtHtt was provided in the Supplemental Figure 1. We corrected the sentence in the introduction as follows in the revised manuscript.

"we recently profiled the proteins that bind to mtHtt on mitochondria and identified valosin-containing-protein (VCP) as a high-abundance mtHtt-interacting protein on mitochondria (Suppl Fig. 1)." (page 3 line 21)

Comment 4: it is not very convincing that VCP interaction is HD-specific - need data from HD patients (HD postmortem brains);

Comment 5: It is important to look at HD postmortem brains for VCP interaction with mHtt in different stages of disease progression.

Response: Following the reviewer's comments 4 and 5, we obtained cortical postmortem brain tissues from five HD patients and five normal subjects from Dr. Eliezer Masliah of the University of California at San Diego. Based on neuropathology reports of these five HD patients, two HD patients (5348 and 5263) exhibited severe brain atrophy and extensive neuronal loss, two HD patients (5298 and 5496) showed moderate brain atrophy and neuronal loss, and one patient (5374) had very subtle neuronal loss. We determined the VCP and mtHtt interactions in total protein lysates of these tissues by immunoprecipitation. We observed the binding between VCP and mtHtt in the four postmortem brain tissues of HD patients who exhibited moderate to severe neuronal loss and brain atrophy (5348, 5263, 5298 and 5496) (new Figure 2D). However, in HD patient with subtle neuronal loss (5374), we did not observe the interaction between VCP and mtHtt (new Suppl Figure 2D). These findings not only support our observation that VCP/mtHtt binding is implicated in HD pathogenesis, but also suggest that the interaction might be relevant to the severity of HD pathology.

In addition, we isolated mitochondria, ER and cytosolic fractions in fibroblasts of two HD patients and two normal subjects, and performed immunoprecipitation to determine the interaction between VCP and mtHtt. Consistent with our findings in HD mouse cells and HD mouse brains, VCP and mtHtt interacted on the mitochondria but not on the ER or cytosolic fractions in HD patient fibroblasts (new Figure 2C). These data from patient cells provided an additional line of evidence to show the relevance of VCP/mtHtt binding to human HD.

The new Figure 2C and 2D, new Supplemental Figure 2D, and related figure legends are added in the revised manuscript. The results on the above data are presented on page 7 line 10 of the revised manuscript. The information for these subjects and the neuropathology evaluation of the five HD patients are summarized in the new Supplemental Fig. 2C.

Reviewer #3 (Remarks to the Author):

Guo and colleagues studied the molecular links between valosin containing protein (VCP) and mitochondria in Huntington's disease (HD) neurons. Authors extensively studied VCP interaction with mutant huntingtin (mHtt), particularly on mitochondria. Authors found VCP translocation to mitochondria in mutant Htt neurons, and facilitate mitochondrial loss through increased mitophagy by recruiting autophagosome component LC3 to mitochondria. They also claimed HV-3 peptide inhibited VCP translocation to mitochondria by reducing mHtt interactions. These observations are interesting. Authors used several reagents, mouse models and progenitor striatal WT (Q7/Q7) and mutant (Q111/Q111) neurons from knockin mice. Authors are well qualified to perform all experiments conducted in this study. Used methods are acceptable. Although VCP is reported to interact with mutant neurodegenerative diseases proteins, including mHtt by several groups (Hirabayashi et al 2001, Almeida et al 2015 and Mori et al 2013) - the present study findings are still novel and interesting for HD and other neurodegenerative disease(s) researchers. If present study findings are reproducible, VCP molecular links with mutant proteins, and their associations with mitophagy are highly significant. Overall, it is a well-done and well executed and well presented manuscript. There are some concerns with the current version:

1) it is important to demonstrate VCP translocation to mitochondria using immunogold electron microscopy;

2) HV-3 peptide story is not convincing at all (it may be worth reporting separately);

3) In the introduction, authors mentioned we recently profiled mitochondrial interactome of mHtt and identified VCP as a high abundance mHtt interacting protein - it is important to show the publication/data;

4) it is not very convincing that VCP interaction is HD-specific - need data from HD patients (HD postmortem brains)

5) It is important to look at HD postmortem brains for VCP interaction with mHtt in different stages of disease progression.

Overall, it is an interesting study - but needs more and solid evidence before publication.

REVIEWERS' COMMENTS:

Reviewer #2 (Remarks to the Author):

The authors have done a great job addressing most, if not all, of the concerns raised by this reviewer. Now the data is of high quality and support their claims. I am still wary of the nature of the HV-3 peptide design, but the evidence is strongly supportive of its property to disrupt Htt-Vcp interaction. There are still some lingering concerns about the possibility of HV-3 interaction with other cellular proteins, which was not fully addressed, and about the lack of proper PK/PD study due to high cost. Taken together, the extensive new data added by the authors and their careful revision of the manuscript strengthen their conclusion on the importance of mHtt-Vcp interaction in HD pathogenesis, hence make it favorable for publication in Nature Communications.

Reviewer #3 (Remarks to the Author):

Authors all the concerns and revised manuscript is improved and ready for publication. Therefore my decision is ACCEPT.

Response letter

RE: VCP recruitment to mitochondria by mutant Huntingtin causes mitophagy impairment and

neurodegeneration in models of Huntington's disease (manuscript reference NCOMMS-16-00149A)

Response: We thank the reviewers for accepting our revised manuscript. We especially would like to thank the reviewers for their critical valuable comments which have helped us to improve our manuscript substantially
